# Supplementary material for: Genomic Analysis of Antimicrobial Resistance Genotype-to-Phenotype Agreement in Helicobacter pylori
Source: Microorganisms. 2020 Dec 22;9(1):2. doi: 10.3390/microorganisms9010002 (PMC7822022; doi:10.3390/microorganisms9010002)
Supplement: Supplementary file 1 [file microorganisms-09-00002-s001.pdf]

Supplementary Material

Genomic Analysis of Antimicrobial Resistance Genotype-to-Phenotype Agreement in *Helicobacter pylori*

Tal Domanovich-Asor[1], Yair Motro[1], Boris Khalfin[1], Hillary A. Craddock[1], Avi Peretz[2] and Jacob Moran-Gilad[1]

[1]MAGICAL Group, Department of Health Systems Management, Faculty of Health Sciences, Ben-Gurion University of the Negev, Beer-Sheva, Israel

[2]Clinical Microbiology Laboratory, Baruch Padeh Medical Center, Poriyah and Azrieli Faculty of Medicine, Bar-Ilan University, Galilee, Israel

Running title: *H. pylori* phenotype-genotype agreement

Address correspondence to:

Prof. Jacob Moran-Gilad, MD MPH

Dept. of Health Systems Management, Faculty of Health Sciences,

Ben Gurion University of the Negev

POB 653, Beer Sheva, 8410501 Israel

Email: giladko@post.bgu.ac.il

Figure S1.

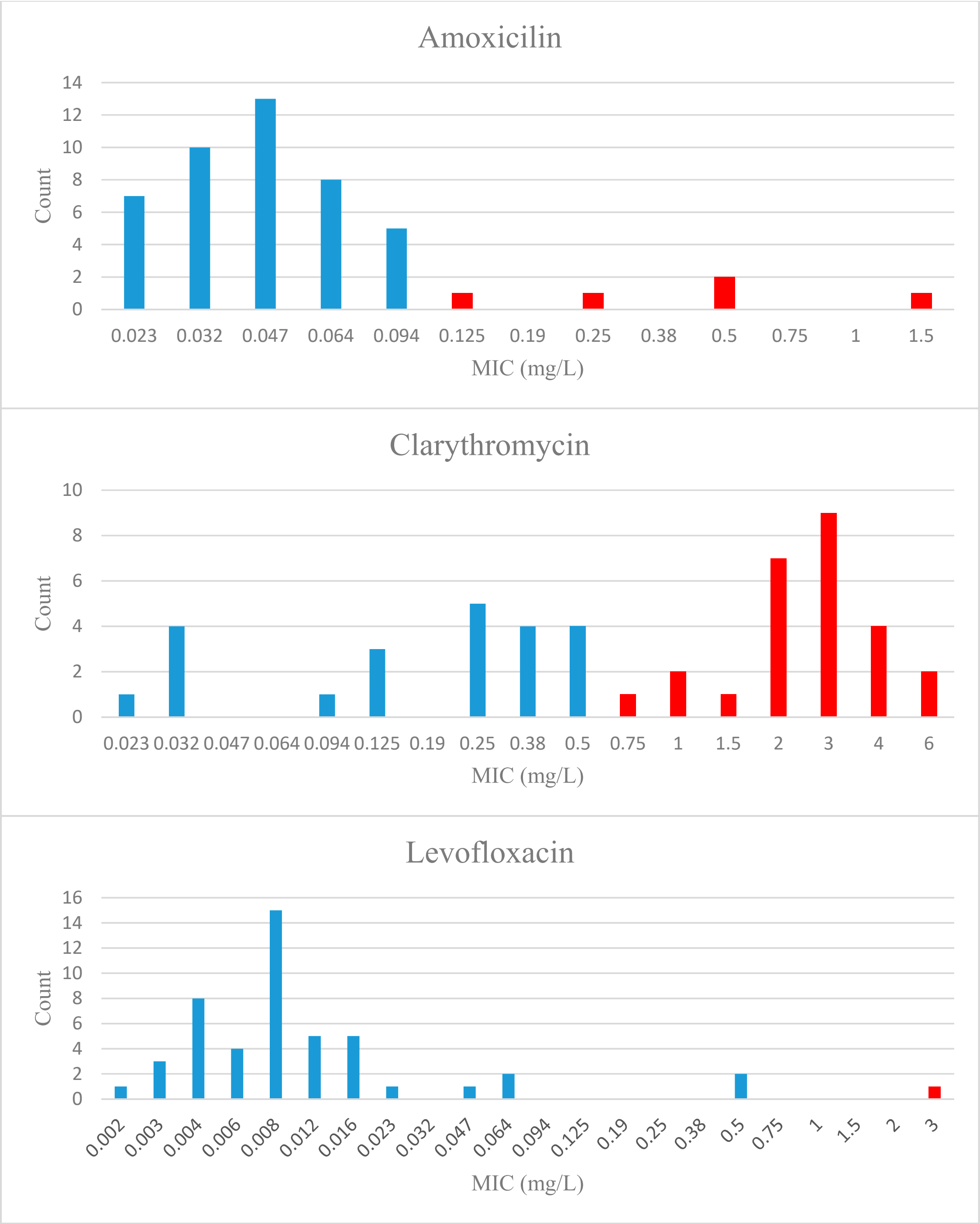
Amoxicilin
Count
14
12
10
8
6
4
2
0
0.023 0.032 0.047 0.064 0.094 0.125 0.19 0.25 0.38 0.5 0.75 1 1.5
MIC (mg/L)
Clarythromycin
Count
10
8
6
4
2
0
0.023 0.032 0.047 0.064 0.094 0.125 0.19 0.25 0.38 0.5 0.75 1 1.5 2 3 4 6
MIC (mg/L)
Levofloxacin
Count
16
14
12
10
8
6
4
2
0
0.002 0.003 0.004 0.006 0.008 0.012 0.016 0.023 0.032 0.047 0.064 0.094 0.125 0.19 0.25 0.38 0.5 0.75 1 1.5 2 3
MIC (mg/L)

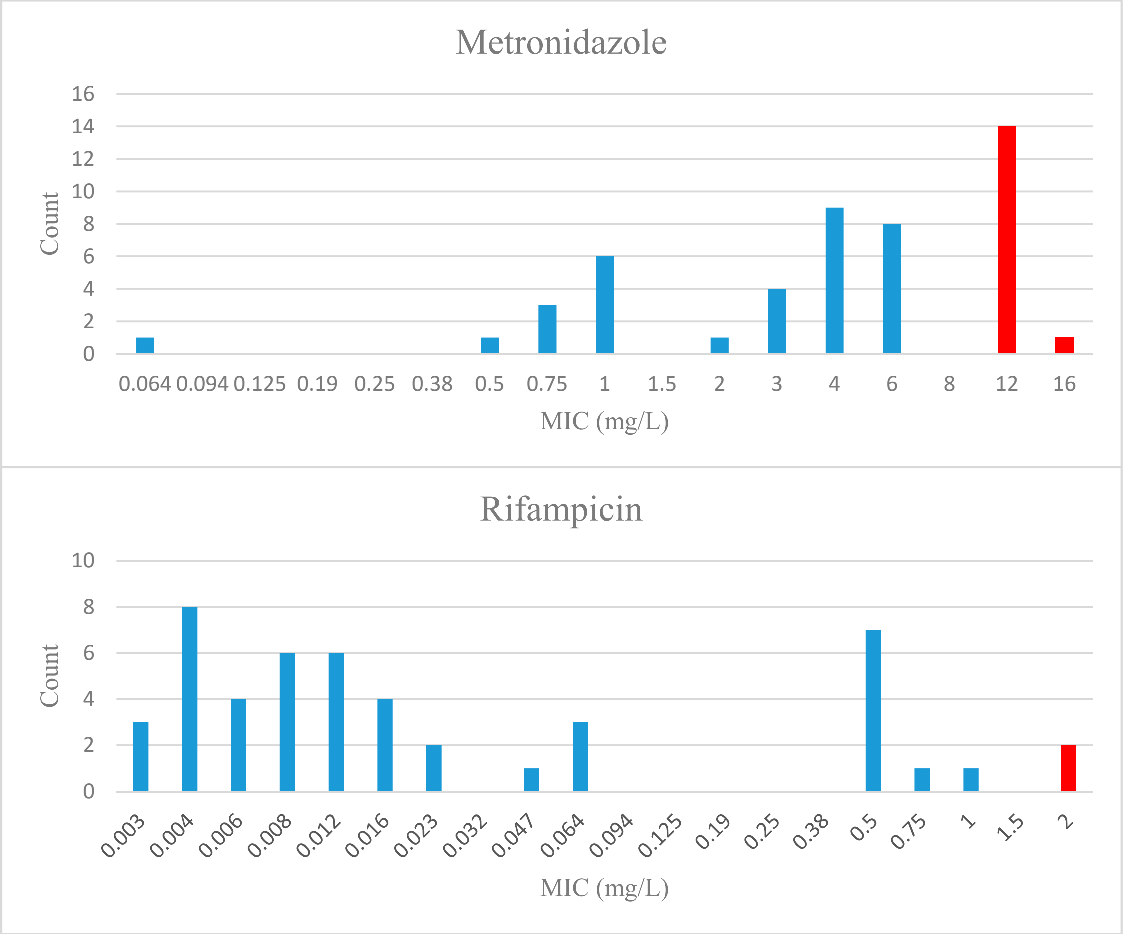

Supplementary Figure 1. Minimum inhibitory concentration (MIC) value distributions for the 48 Israeli isolates for amoxicillin, clarithromycin, levofloxacin, metronidazole, and rifampicin. Blue bars indicate MIC values which render the isolate susceptible to the particular agent, whereas red bars indicate resistant isolates per BSAC breakpoints.

Supplementary Table 2. List of previously described mutations associated with phenotypic resistance in *H. pylori* and mutations observed for the first time in this study.

Antimicrobial	Gene	Mutation	Reference
AMO	*pbp1A*	A69V	1
		S414R	
		Y484C	
		T541I	
		T556S	
		N562Y	1,2
		S402G	3
		E406A	
		S543R	
		S589G	
		G595S	
		S417T	
		A369T	4
		V374L	
		L423E	
		T593A	
		G94E	This study
		N461D	
CLA	*23S rRNA*	G2141A	5
		A2142G\C	
		A2143G\C\T	
		G2224A	6
		T2182C	7
		T2190C	
		C2195T	
		A2223G	
		G2111A	8
		A2115G	
		A2144G	
		C2147G	
		G2172T	9
		G2254T	
		C2173T	This study
		G2212A	
LEV	*gyrA*	D91Y\G\N	10
		A97V	
		A88V	
		N87K\I\Y	10,11
		D86N	11
		R140K	12

		D192N	
		M191I	13
		V199A	
		G208E	
		I234V	
		A272G	
		H354R	
		R397Q	
		N495S	
		I498T	
		Y513H	
		A524V	
		D610N	
		E632G	
		S633G	
		S652G	
		E679D	
		K694R	
		G733E	
		M803V	
		D143E	14
		A207T	
		E214K	
		T239M	This study
MET	*rdxA*	H25R	13
		S30R	
		P44L	
		S45R	
		V111A	
		A183V	
		L185Y	
		L188V	
		Y47C	15
		K63E	
		A143T	
		G145V	
		R200G	
		P51L	16
		A67V	
		D157N	17
		G189C	17,18
		T49K	19
		Y26V	20
		A37V	

		A40P	
		K64N	
		R90K	
		L153-stop	
		V172I	
		Q50-stop	20,21
		R16H\C	18
		C19Y	
		T31E	
		H97Y\T	
		S108A	
		A118T/s	
		R131K	
		D59N	21
		V204I	
		A206T	
		V86I	This study
		P96L	
		G122R	
	frxA	A32V	20
		W68-stop	
		A152V	
		A153V	
		F72S	21
		G73S	
		C193S	
		A70T	This study
		A138V	

RIF	*rpoB*	L525P	22
		S545L	
		I586L\N	
		V149F	23
		Q527R\K	24
		D530V/Y/G/F	
		H540N\Y	25
		R701H	
		L547F	26
		V783M	This study

additional variants references; 27,28,29,30,31,32,33,34,35,36,37

Supplementary Table 3. Genotype to phenotype concordance of Israeli isolates (n=48) with respect to published mutations known to confer resistance and novel point mutations

	pbp1A		23s *rRNA*		*gyrA*		*rdxA*		*frxA*		*rpoB*	
All perfectly aligned Israeli isolates / MIC values	Isolates	Isolates with novel mutations	Isolates	Isolates with novel mutations	Isolates	Isolates with novel mutations	Isolates	Isolates with novel mutations	Isolates	Isolates with novel mutations	Isolates	Isolates with novel mutations
16	-	-	-	-	-	-	**1**	-	**1**	-	-	-
12	-	-	-	-	-	-	**10**	**3** (V86I, G122R, P96L)	**8**	**2** (A70T, A138V)	-	-
6	-	-	**1**	1 (G2212A)	-	-	6	1 (V86I)	6	-	-	-
4	-	-	**3**	-	-	-	7	1 (P96L)	5	-	-	-
3	-	-	**8**	1 (C2173T)	-	**1** (T239M)	4	-	4	-	-	-
2	-	-	**5**	1 (G2212A)	-	-	1	-	-	-	**1**	**1** (D530V, R701H)
1.5	-	**1** (G94E N461D)	**1**	-	-	-	-	-	-	-	-	-
1	-	-	**2**	-	-	-	4	-	1	-	1	-
0.75	-	-	1	-	-	-	3	-	1	-	1	-
0.5	**2***	-	4	-	2	-	1	-	1	-	7	-
0.38	-	-	4	-	-	-	-	-	-	-	-	-
0.25	-	-	4	-	-	-	-	-	-	-	-	-
0.125	-	-	3	-	-	-	-	-	-	-	-	-
0.094	4	-	1	-	-	-	-	-	-	-	-	-
0.064	6	1 (N461D)	-	-	2	-	1	-	1	-	3	-
0.047	12	-	-	-	1	-	-	-	-	-	1	-

0.032	6	-	4	-	-	-	-	-	-	-	-	-
0.023	5	-	-	1 (G2212A)	1	-	-	-	-	-	2	-
0.016	-	-	-	-	5	-	-	-	-	-	4	-
0.012	-	-	-	-	5	-	-	-	-	-	4	1 (V783M)
0.008	-	-	-	-	14	-	-	-	-	-	6	-
0.006	-	-	-	-	4	-	-	-	-	-	4	-
0.004	-	-	-	-	8	-	-	-	-	-	-	-
0.003	-	-	-	-	3	-	-	-	-	-	-	-
0.002	-	-	-	-	1	-	-	-	-	-	-	-
SUM	35	2	41	4	46	1	38	5	28	2	34	1

*Highlighted numbers in bold refers to phenotypically resistant isolates according to BSAC guidelines.

Supplementary Table 4. Mutations observed in the *pbp1A* gene (previously described* and novel mutations). After imperfect alignments were omitted, 37 of the total 48 sequences remained for analysis.

Mutation	WT	Published mutations		New variant	New mutation	Reference
		Likely associated with resistance phenotype	Additional variants of the mutation			
A69V	A<br>37	V<br>0				1
A369T	A<br>36	T<br>1				3
V374L	V<br>37	L<br>0				3
S402G	S<br>19	G<br>0		I<br>0		2
E406A	E<br>4	A<br>32	K V T<br>0 0 1	M Q<br>0 0		2
S414R	S<br>36	R<br>0		N<br>1		1
S417T	S<br>35	T<br>2				2
L423E	L<br>37	E<br>0				3
Y484C	Y<br>37	C<br>0				1
T541I	T<br>37	I<br>0				1
S543R	S<br>15	R<br>20	H N T<br>2 0 0			2
T556S	T<br>37	S<br>0				1
N562Y	N<br>34	Y<br>3	D<br>0	H G<br>0 0		1, 2
S589G	S	G		D		2

	21	16		0		
T593A**	T<br>14	A<br>20	G<br>0	S P<br>2 0		3
G595S	G<br>8	S<br>25		A<br>4		2
G94E	G<br>35				E<br>1	This study
N461D	N<br>32				D<br>5	This study

*Observed variants not present in the mutation library were searched against the NCBI database to determine if they were previously described (but not associated with resistance), and if found in NCBI they were described as “additional variants of the mutation”. Variants that were not detected previously in literature in the context of antibiotic resistance were referred to as “new variants.”

**36 out of 48 alignments were used in the analysis of this mutation

Supplementary Table 5. Mutations observed in the 23s *rRNA* gene (previously described in the literature* and novel mutations). After imperfect alignments were omitted, 45 of the total 48 sequences remained for analysis.

Mutation	WT	Published mutations<br>Likely associated with resistance phenotype	New mutation	Reference
G2111A	G<br>45	A<br>0		8
A2115G	A<br>45	G<br>0		8
G2141A	G<br>45	A<br>0		5
A2142G\C	A<br>44	G C<br>1 0		5
A2143G\C\T	A<br>28	G C T<br>17 0 0		5
A2144G	A<br>45	G<br>0		8
C2147G	C<br>45	G<br>0		8
G2172T	G<br>45	T<br>0		9
T2182C	T<br>43	C<br>2		7
T2190C	T<br>45	C<br>0		7
C2195T	C<br>45	T<br>0		7
A2223G	A<br>45	G<br>0		7
G2224A	G<br>45	A<br>0		6
G2254T	G	T		9

	45	0		
C2173T	C		T	This study
	44		1	
G2212A	G		A	This study
	42		3	

*Observed variants not present in the mutation library were searched against the NCBI database to determine if they were previously described (but not associated with resistance), and if found in NCBI they were described as "additional variants of the mutation". Variants that were not detected previously in literature in the context of antibiotic resistance were referred to as "new variants."

Supplementary Table 6. Mutations observed in the *gyrA* gene (previously described* and novel mutations). After imperfect alignments were omitted, 47 of the total 48 sequences remained for analysis.

Mutation	WT	Published mutations		New variant	New mutation	Reference
		Likely associated with resistance phenotype	Additional variants of the mutation			
D86N	D<br>47	N<br>0				11
N87K\I\Y	N<br>43	K I Y<br>2 1 0	T<br>1	S H<br>0 0		10, 11
A88V	A<br>47	V<br>0	P<br>0			10
D91Y\G\N	D<br>45	Y G N<br>0 0 2				10
A97V	A<br>47	V<br>0				10
R140K	R<br>46	K<br>1				12
D143E	D<br>47	E<br>0		N<br>0		14
M191I	M<br>8	I<br>39		F T<br>0 0		13
D192N	D<br>45	N<br>2				12
V199A	V<br>37	A<br>10	I<br>0	M T E<br>0 0 0		13
A207T	A<br>46	T<br>1				14
G208E	G<br>1	E<br>35	A<br>11	R K Q<br>0 0 0		13
E214K	E<br>47	K<br>0		G<br>0		14
I234V	I	V				13

	37	10				
A272G	A<br>47	g<br>0				13
H354R	H<br>1	R<br>43		C S<br>3 0		13
R397Q	R<br>16	Q<br>31				13
N495S	N<br>35	S<br>12		G<br>0		13
I498T	I<br>35	T<br>12		M<br>0		13
Y513H	Y<br>44	H<br>3				13
A524V	A<br>44	V<br>3				13
D610N	D<br>47	N<br>0				13
E632G	E<br>32	G<br>15		D S<br>0 0		13
S633G	S<br>37	G<br>10				13
S652G	S<br>35	G<br>12		D T<br>0 0		13
E679D	E<br>5	D<br>42		N Y<br>0 0		13
K694R	K<br>27	R<br>20		S<br>0		13
G733E	G<br>1	E<br>46				13
M803V	M<br>35	V<br>12		K I<br>0 0		13
T239M	T<br>46				M<br>1	This study

*Observed variants not present in the mutation library were searched against the NCBI database to determine if they were previously described (but not associated with resistance), and if found in NCBI they were described as “additional variants of the mutation”. Variants that were not detected previously in literature in the context of antibiotic resistance were referred to as “new variants.”

Supplemental Table 7. Mutations observed in the *rdxA* gene (previously described* and novel mutations). Number of alignments remaining after imperfect alignments were removed is represented in the "N" column.

Mutation	N	WT	Published mutations		New variant		New mutation	Reference
			Likely associated with resistance phenotype	Additional variants of the mutation				
R16H\C	43	R<br>41	H C<br>1 1					18
C19Y	43	C<br>34	Y<br>0		F<br>0			18
H25R	43	H<br>42	R<br>1		Y<br>0			13
Y26V	43	Y<br>43	V<br>0					20
S30R	43	S<br>43	R<br>0		N<br>0			13
T31E	43	T<br>37	E<br>6	A<br>0	K<br>0	G<br>0		18
A37V	43	A<br>43	V<br>0		S<br>0	T<br>0		20
A40P	43	A<br>43	p<br>0		T<br>0			20
P44L	43	P<br>43	L<br>0					13
S45R	43	S<br>43	R<br>0		N<br>0			13
Y47C	43	Y<br>43	C<br>0					15
T49K	43	T<br>43	K<br>0					19
Q50-stop	43	Q<br>43	stop<br>0					20, 21
P51L		P	L					39

	43	43	0								
D59N		D	N				S				21
	43	0	43				0				
K63E		K	E		Q						15
	43	43	0		0						
K64N		K	N				R	S			20
	43	43	0				0	0			
A67V		A	V								13
	43	42	1								
R90K		R	K				N	G	S		20
	43	28	14				0	0	1		
H97Y\T		H	T	Y			I	M			18
	43	34	6	3			0	0			
S108A		S	A				P	T			18
	43	41	2				0	0			
V111A		V	A				M				13
	43	38	5				0				
A118T/s		A	T	S							18
	43	40	3	0							
R131K		R	K								18
	43	24	19								
A143T		A	T								15
	43	43	0								
G145V		G	V		E	R	W	A			15
	43	43	0		0	0	0	0			
L153-stop		L	stop								20
	43	43	0								
D157N		D	N				Y	Q	G		20
	43	43	0				0	0	0		
V172I		V	I								20
	43	38	5								
A183V		A	V				R				13
	41	37	4				0				
L185Y		L	Y				K				13
	41	41	0				0				
L188V		L	V				R	F			13

	41	41	0		0 0		
G189C	41	G<br>41	C<br>0		D I S<br>0 0 0		17, 18
R200G	41	R<br>41	G<br>0		E D<br>0 0		15
V204I	41	V<br>25	I<br>16		L Q A T<br>0 0 0 0		21
A206T	41	A<br>41	T<br>0		Q K L E<br>0 0 0 0		21
V86I	43	V<br>41				I<br>2	This study
P96L	43	P<br>41				L<br>2	This study
G122R	43	G<br>40			S<br>2	R<br>1	This study

*Observed variants not present in the mutation library were searched against the NCBI database to determine if they were previously described (but not associated with resistance), and if found in NCBI they were described as "additional variants of the mutation". Variants that were not detected previously in literature in the context of antibiotic resistance were referred to as "new variants."

Supplementary Table 8. Mutations observed in the *frxA* gene (previously described* and novel mutations). After imperfect alignments were omitted, 30 of the total 48 sequences remained for analysis.

Mutation	WT	Published mutations<br>Likely associated with resistance phenotype	New variant	New mutations	Reference
A32V	A<br>30	V<br>0	T S<br>0 0		20
W68-stop	W<br>30	stop<br>0			20
F72S	F<br>30	S<br>0			21
G73S	G<br>30	S<br>0			21
A152V	A<br>28	V<br>2	T<br>0		20
A153V	A<br>30	V<br>0	G Q T<br>0 0 0		20
C193S	C<br>30	S<br>0	F V<br>0 0		21
A70T			A G V<br>28 0 0	T<br>2	This study
A138V			A T<br>28 0	V<br>2	This study

*Observed variants not present in the mutation library were searched against the NCBI database to determine if they were previously described (but not associated with resistance), and if found in NCBI they were described as “additional variants of the mutation”. Variants that were not detected previously in literature in the context of antibiotic resistance were referred to as “new variants.”

Supplementary Table 9. Mutations observed in the *rpoB* gene (previously described* and novel mutations). After imperfect alignments were omitted, 46 of the total 48 sequences remained for analysis.

Mutation	WT	Published mutations		New variant	New mutation	Reference
		Likely associated with resistance phenotype	Additional variants of the mutation			
V149F	V<br>46	F<br>0				23
L525P	L<br>46	P<br>0				22
Q527R\K	Q<br>46	R<br>0				24
D530V	D<br>45	V<br>1	N E<br>0 0			25
H540N\Y	H<br>46	N<br>0				25
S545L	S<br>46	L<br>0				22
L547F**	L<br>45	F<br>0				26
I586L\N	I<br>46	L N<br>0 0				22
R701H	R<br>45	H<br>1		C<br>0		25
V783M	V<br>44				M<br>2	This study

*Observed variants not present in the mutation library were searched against the NCBI database to determine if they were previously described (but not associated with resistance), and if found in NCBI they were described as “additional variants of the mutation”. Variants that were not detected previously in literature in the context of antibiotic resistance were referred to as “new variants.”

** 45 out of 48 alignments were used in the analysis of this mutation.

References.

1. Okamoto, T.; Yoshiyama, H.; Nakazawa, T.; Park, I.-D.; Chang, M.-W.; Yanai, H.; Okita, K.; Shirai, M. A change in PBP1 is involved in amoxicillin resistance of clinical isolates of Helicobacter pylori. *J. Antimicrob. Chemother.* **2002**, *50*, 849–856, doi:10.1093/jac/dkf140.
2. Rimbara, E.; Noguchi, N.; Kawai, T.; Sasatsu, M. Mutations in penicillin-binding proteins 1, 2 and 3 are responsible for amoxicillin resistance in Helicobacter pylori. *J. Antimicrob. Chemother.* **2008**, *61*, 995–998, doi:10.1093/jac/dkn051.
3. Gerrits, M.M.; Godoy, A.P.O.; Kuipers, E.J.; Ribeiro, M.L.; Stoof, J.; Mendonça, S.; Van Vliet, A.H.M.; Jr, J.P.; Kusters, J.G. Multiple Mutations in or Adjacent to the Conserved Penicillin-Binding Protein Motifs of the Penicillin-Binding Protein 1A Confer Amoxicillin Resistance to Helicobacter pylori. *Helicobacter* **2006**, *11*, 181–187, doi:10.1111/j.1523-5378.2006.00398.x.
4. Rimbara, E.; Noguchi, N.; Kawai, T.; Sasatsu, M. Correlation between Substitutions in Penicillin-Binding Protein 1 and Amoxicillin Resistance inHelicobacter pylori. *Microbiol. Immunol.* **2007**, *51*, 939–944, doi:10.1111/j.1348-0421.2007.tb03990.x.
5. Van Doorn, L.-J.; Debets-Ossenkopp, Y.J.; Marais, A.; Sanna, R.; Megraud, F.; Kusters, J.G.; Quint, W.G.V. Rapid Detection, by PCR and Reverse Hybridization, of Mutations in the Helicobacter pylori 23S rRNA Gene, Associated with Macrolide Resistance. *Antimicrob. Agents Chemother.* **1999**, *43*, 1779–1782, doi:10.1128/aac.43.7.1779.
6. Hao, Q.; Li, Y.; Zhang, Z.J.; Liu, Y.; Gao, H. New mutation points in 23S rRNA gene associated with Helicobacter pylori resistance to clarithromycin in northeast China. World journal of gastroenterology. 2004, 10,1075.
7. Kim, J.M.; Kim, J.S.; Kim, N.; Kim, Y.-J.; Kim, I.Y.; Chee, Y.J.; Lee, C.-H.; Jung, H.C. Gene mutations of 23S rRNA associated with clarithromycin resistance in Helicobacter pylori strains isolated from Korean patients. *J. Microbiol. Biotechnol.* **2008**, *18*, 1584–1589.
8. Hansomburana, P.; Anantapanpong, S.; Sirinthornpunya, S.; Chuengyong, K.; Rojborwonwittaya, J. Prevalence of single nucleotide mutation in clarithromycin resistant gene of Helicobacter pylori: A 32-months prospective study by using hybridization real time polymerase chain reaction. *J. Med Assoc. Thail. = Chotmaihet thangphaet* 2012, *95*.
9. Zhen-Hua, Z.; De-Qiang, H.; Yong, X.; Lin-Lin, L.; Nong-Hua, L. Characterization of 23S rRNA gene mutation in primary and secondary clarithromycin-resistant Helicobacter pylori strains from East China. *Turk. J. Gastroenterol.* **2009**, *24*, 5–9, doi:10.4318/tjg.2013.0525.
10. Moore, R.A.; Beckthold, B.; Wong, S.; Kureishi, A.; Bryan, L.E. Nucleotide sequence of the gyrA gene and characterization of ciprofloxacin-resistant mutants of Helicobacter pylori. *Antimicrob. Agents Chemother.* **1995**, *39*, 107–111, doi:10.1128/aac.39.1.107.
11. Cattoir, V.; Nectoux, J.; Lascols, C.; Deforges, L.; Delchier, J.-C.; Mégraud, F.; Soussy, C.-J.; Cambau, E. Update on fluoroquinolone resistance in Helicobacter pylori: New mutations leading to resistance and first description of a gyrA polymorphism associated with hypersusceptibility. *Int. J. Antimicrob. Agents* **2007**, *29*, 389–396, doi:10.1016/j.ijantimicag.2006.11.007.
12. Miftahussurur, M.; Syam, A.F.; Nusi, I.A.; Makmun, D.; Waskito, L.A.; Zein, L.H.; Akil, F.; Uwan, W.B.; Simanjuntak, D.; Wibawa, I.D.N.; et al. Surveillance of Helicobacter pylori Antibiotic Susceptibility in Indonesia: Different Resistance Types among Regions and with Novel Genetic Mutations. *PLoS ONE* **2016**, *11*, e0166199, doi:10.1371/journal.pone.0166199.
13. Lauener FN, Imkamp F, Lehours P, Buissonnière A, Benejat L, Zbinden, R.; et al. Genetic determinants and prediction of antibiotic resistance phenotypes in Helicobacter pylori. Journal of clinical medicine. 2019, *8*, 53.
14. Farzi, N.; Yadegar, A.; Sadeghi, A.; Aghdaei, H.A.; Smith, S.M.; Raymond, J.; Suzuki, H.; Zali, M.R. High Prevalence of Antibiotic Resistance in Iranian Helicobacter pylori Isolates: Importance of Functional and Mutational Analysis of Resistance Genes and Virulence Genotyping. *J. Clin. Med.* **2019**, *8*, 2004, doi:10.3390/jcm8112004.
15. Goodwin, A.; Kersulyte, D.; Sisson, G.; Van Zanten, S.J.O.V.; Berg, D.E.; Hoffman, P.S. Metronidazole resistance in Helicobacter pylori is due to null mutations in a gene (rdxA) that encodes an oxygen-insensitive NADPH nitroreductase. *Mol. Microbiol.* **1998**, *28*, 383–393, doi:10.1046/j.1365-2958.1998.00806.x.
16. Jenks, P.J.; Ferrero, R.L.; Labigne, A. The role of the rdxA gene in the evolution of metronidazole resistance in Helicobacter pylori. Journal of Antimicrobial Chemotherapy. 1999, 43,753–758.
17. Kwon, D.H.; Hulten, K.; Kato, M.; Kim, J.J.; Lee, M.; El-Zaatari, F.A.K.; Osato, M.S.; Graham, D.Y. DNA Sequence Analysis of rdxA andfrxA from 12 Pairs of Metronidazole-Sensitive and -Resistant Clinical Helicobacter pyloriIsolates. *Antimicrob. Agents Chemother.* **2001**, *45*, 2609–2615, doi:10.1128/aac.45.9.2609-2615.2001.
18. Rasheed, F.; Campbell, B.J.; Alfizah, H.; Varro, A.; Zahra, R.; Yamaoka, Y.; et al. Analysis of clinical isolates of Helicobacter pylori in Pakistan reveals high degrees of pathogenicity and high frequencies of antibiotic resistance. Helicobacter. 2014, 19,387–399.
19. Paul, R.; Postius, S.; Melchers, K.; Scha¨ferK.P. Mutations of the Helicobacter pyloriGenes rdxA and pbp1 Cause Resistance against Metronidazole and Amoxicillin. *Antimicrob. Agents Chemother.* **2001**, *45*, 962–965, doi:10.1128/aac.45.3.962-965.2001.
20. Kim, S.Y.; Joo, Y.M.; Lee, H.S.; Chung, I.-S.; Yoo, Y.-J.; Merrell, D.S.; Cha, J.-H. Genetic analysis of Helicobacter pylori clinical isolates suggests resistance to metronidazole can occur without the loss of functional rdxA. *J. Antibiot.* **2009**, *62*, 43–50, doi:10.1038/ja.2008.6.
21. Butlop, T.; Mungkote, N.; Chaichanawongsaroj, N. Analysis of allelic variants of rdxA associated with metronidazole resistance in Helicobacter pylori: Detection of common genotypes in rdxA by multiplex allele-specific polymerase chain reaction. *Genet. Mol. Res.* **2016**, *15*, 3, doi:10.4238/gmr.15038674.
22. Heep, M.; Beck, D.; Bayerdo¨rfferE.; Lehn, N. Rifampin and Rifabutin Resistance Mechanism inHelicobacter pylori. *Antimicrob. Agents Chemother.* **1999**, *43*, 1497–1499, doi:10.1128/aac.43.6.1497.

23. Heep, M.; Rieger, U.; Beck, D.; Lehn, N. Mutations in the beginning of the rpoB Gene can induce resistance to Rifamycins in both helicobacter pylori and mycobacterium tuberculosis. Antimicrobial agents and chemotherapy. 2000, *44*, 1075–1077.
24. Glocker, E.; Bogdan, C.; Kist, M. Characterization of rifampicin-resistant clinical Helicobacter pylori isolates from Germany. *J. Antimicrob. Chemother.* **2007**, *59*, 874–879, doi:10.1093/jac/dkm039.
25. Heep, M.; Odenbreit, S.; Beck, D.; Decker, J.; Prohaska, E.; Rieger, U.; Lehn, N. Mutations at Four Distinct Regions of the rpoB Gene Can Reduce the Susceptibility of Helicobacter pylorito Rifamycins. *Antimicrob. Agents Chemother.* **2000**, *44*, 1713–1715, doi:10.1128/aac.44.6.1713-1715.2000.
26. Hays, C.; Burucoa, C.; Lehours, P.; Tran, C.T.; Leleu, A.; Raymond, J. Molecular characterization of Helicobacter pylori resistance to rifamycins. *Helicobacter*. 2018, *23*, e12451.
27. Kageyama, C.; Sato, M.; Sakae, H.; Obayashi, Y.; Kawahara, Y.; Mima, T.; Matsushita, O.; Yokota, K.; Mizuno, M.; Okada, H. Increase in antibiotic resistant Helicobacter pylori in a University Hospital in Japan. *Infect. Drug Resist.* **2019**, *12*, 597–602, doi:10.2147/idr.s196452.
28. Arévalo, A.; Otero, W.; Trespalacios, A.A. Helicobacter pylori: Resistencia múltiple en pacientes de Bogotá, Colombia. *Biomédica*. **2019**, *39*, 125–134.
29. De Palma, G.Z.; Mendiondo, N.; Wonaga, A.; Viola, L.; Ibarra, D.; Campitelli, E.; Salim, N.; Corti, R.; Goldman, C.; Catalano, M. Occurrence of Mutations in the Antimicrobial Target Genes Related to Levofloxacin, Clarithromycin, and Amoxicillin Resistance inHelicobacter pyloriIsolates from Buenos Aires City. *Microb. Drug Resist.* **2017**, *23*, 351–358, doi:10.1089/mdr.2015.0361.
30. Eng, N.F.; Ybazeta, G.; Chapman, K.; Fraleigh, N.L.; Letto, R.; Altman, E.; Diaz-Mitoma, F. Antimicrobial susceptibility of Canadian isolates of Helicobacter pylori in Northeastern Ontario. *Can. J. Infect. Dis. Med. Microbiol.* **2015**, *26*, 137–144.
31. Bachir, M.; Allem, R.; Benejat, L.; Tifrit, A.; Medjekane, M.; Drici, A.E.-M.; Megraud, F.; Douidi, K.T. Molecular detection of mutations involved in Helicobacter pylori antibiotic resistance in Algeria. *J. Antimicrob. Chemother.* **2018**, *73*, 2034–2038, doi:10.1093/jac/dky167.
32. Tankovic, J.; Lascols, C.; Sculo, Q.; Petit, J.-C.; Soussy, C.-J. Single and Double Mutations in gyrA but Not in gyrB Are Associated with Low- and High-Level Fluoroquinolone Resistance in Helicobacter pylori. *Antimicrob. Agents Chemother.* **2003**, *47*, 3942–3944, doi:10.1128/aac.47.12.3942-3944.2003.
33. Secka O, Berg DE, Antonio M, Corrah T, Tapgun M, Walton R,et al. Antimicrobial susceptibility and resistance patterns among Helicobacter pylori strains from The Gambia, West Africa. Antimicrob. agents Chemother. . 2013, *57*, 1231–1237.
34. Teh, X.; Khosravi, Y.; Lee, W.C.; Leow, A.H.R.; Loke, M.F.; Vadivelu, J.; Goh, K.L. Functional and Molecular Surveillance of Helicobacter pylori Antibiotic Resistance in Kuala Lumpur. *PLoS ONE* **2014**, *9*, e101481, doi:10.1371/journal.pone.0101481.
35. Miftahussurur, M.; Cruz, M.; Subsomwong, P.; Abreu, J.A.J.; Hosking, C.; Nagashima, H.; Akada, J.; Yamaoka, Y. Clarithromycin-Based Triple Therapy is Still Useful as an Initial Treatment for Helicobacter pylori Infection in the Dominican Republic. *Am. J. Trop. Med. Hyg.* **2017**, *96*, 1050–1059, doi:10.4269/ajtmh.16-0729.
36. Heep M, Lehn N, Brandstätter B, Rieger U, Senzenberger, S.; Wehrl, W. Detection of rifabutin resistance and association of rpoB mutations with resistance to four rifamycin derivatives in Helicobacter pylori. *Eur. J. Clin. Microbiol. Infect. Dis*. 2002, *21*,143.
37. Mori, H.; Suzuki, H.; Matsuzaki, J.; Masaoka, T.; Kanai, T. Acquisition of double mutation in gyrA caused high resistance to sitafloxacin in Helicobacter pylori after unsuccessful eradication with sitafloxacin-containing regimens. *United Eur. Gastroenterol. J.* **2018**, *6*, 391–397, doi:10.1177/2050640617737215.
